# Supplementary material for: Context-dependent modulations of subthalamo-cortical synchronization during rapid reversals of movement direction in Parkinson’s disease
Source: eLife. 2025 Jun 5;13:RP101769. doi: 10.7554/eLife.101769 (PMC12140627; doi:10.7554/eLife.101769)
Supplement: Supplementary file 1. — (A) Effects of condition (predictable, unpredictable) and movement (start, reverse, stop) on movement-aligned speed, controlling for age, pre-operative UPDRS score, and disease duration. (B) Effects of condition (predictable, unpredictable) and movement (start, reverse, stop) on reaction times to cues, controlling for age, pre-operative UPDRS score, and disease duration. [file elife-101769-supp1.docx]

Supplementary File 1: Behavioral effects. (A) Effects of condition (predictable, unpredictable) and movement (start, reverse, stop) on movement-aligned speed, controlling for age, pre-operative UPDRS score and disease duration. (B) Effects of condition (predictable, unpredictable) and movement (start, reverse, stop) on reaction times to cues, controlling for age, pre-operative UPDRS score and disease duration.

**A**

| Factor | Wilk’s Lambda | *F* | Hypothesis *df* | Error *df* | Sig. | η_p_^2^ |
| --- | --- | --- | --- | --- | --- | --- |
| Condition | 0.998 | 0.037 | 1 | 16 | 0.850 | 0.002 |
| Condition*age | 0.974 | 0.425 | 1 | 16 | 0.524 | 0.026 |
| Condition*UPDRS | 1,000 | 0.002 | 1 | 16 | 0.966 | 0,000 |
| Condition*disease duration | 0.894 | 1.897 | 1 | 16 | 0.187 | 0.106 |
| Movement | **0.412** | **10.695** | **2** | **15** | **0.001** | **0.588** |
| Movement*age | 0.987 | 0.098 | 2 | 15 | 0.908 | 0.013 |
| Movement*UPDRS | 0.971 | 0.223 | 2 | 15 | 0.803 | 0.029 |
| Movement*disease duration | 0.992 | 0.059 | 2 | 15 | 0.943 | 0.008 |
| Condition*movement | 0.762 | 2.345 | 2 | 15 | 0.130 | 0.238 |
| Condition*movement*age | 0.983 | 0.129 | 2 | 15 | 0.880 | 0.017 |
| Condition*movement*UPDRS | 0.848 | 1.344 | 2 | 15 | 0.291 | 0.152 |
| Condition*movement*disease duration | 0.939 | 0.483 | 2 | 15 | 0.626 | 0.061 |

**B**

| Condition | 0.705 | 6.698 | 1 | 16 | 0.020 | 0.295 |
| --- | --- | --- | --- | --- | --- | --- |
| Condition*age | 0.987 | 0.205 | 1 | 16 | 0.657 | 0.013 |
| Condition*UPDRS | 0.967 | 0.544 | 1 | 16 | 0.472 | 0.033 |
| Condition*disease duration | 0.998 | 0.031 | 1 | 16 | 0.862 | 0.002 |
| Movement | **0.278** | **19.482** | **2** | **15** | **<0.001** | **0.722** |
| Movement*age | 0.968 | 0.251 | 2 | 15 | 0.781 | 0.032 |
| Movement*UPDRS | 0.906 | 0.780 | 2 | 15 | 0.476 | 0.094 |
| Movement*disease duration | 0.815 | 1.708 | 2 | 15 | 0.215 | 0.185 |
| Condition*movement | **0.604** | **4.916** | **2** | **15** | **0.023** | **0.396** |
| Condition*movement*age | 0.828 | 1.556 | 2 | 15 | 0.243 | 0.172 |
| Condition*movement* UPDRS | 0.881 | 1.015 | 2 | 15 | 0.386 | 0.119 |
| Condition*movement* disease duration | 0.900 | 0.832 | 2 | 15 | 0.454 | 0.100 |
