# Supplementary material for: Context-dependent modulations of subthalamo-cortical synchronization during rapid reversals of movement direction in Parkinson’s disease
Source: eLife. 2025 Jun 5;13:RP101769. doi: 10.7554/eLife.101769 (PMC12140627; doi:10.7554/eLife.101769)
Supplement: Supplementary file 2. — Effects of modulation type (beta suppression, beta rebound), condition (predictable, unpredictable), and ROI (STN, M1, MSMC) on lateralization index, controlling for age, pre-operative UPDRS score, and disease duration. [file elife-101769-supp2.docx]

Supplementary File 2: Effects on lateralization. Effects of modulation type (beta suppression, beta rebound), condition (predictable, unpredictable) and ROI (STN, M1, MSMC) on lateralization index, controlling for age, pre-operative UPDRS score and disease duration.

| Factor | Wilk’s Lambda | *F* | Hypothesis *df* | Error *df* | Sig. | η_p_^2^ |
| --- | --- | --- | --- | --- | --- | --- |
| Modulation type | **0.467** | **18.233** | **1** | **16** | **<0.001** | **0.533** |
| Modulation type*age | 0.989 | 0.185 | 1 | 16 | 0.673 | 0.011 |
| Modulation type*UPDRS | 0.987 | 0.213 | 1 | 16 | 0.651 | 0.013 |
| Modulation type*disease duration | 0.892 | 1.931 | 1 | 16 | 0.184 | 0.108 |
| ROI | **0.597** | **5.071** | **2** | **15** | **0.021** | **0.403** |
| RO*age | 0.960 | 0.313 | 2 | 15 | 0.736 | 0.040 |
| ROI*UPDRS | 0.861 | 1.210 | 2 | 15 | 0.326 | 0.139 |
| ROI*disease duration | 0.921 | 0.639 | 2 | 15 | 0.542 | 0.079 |
| Condition | 0.950 | 0.836 | 1 | 16 | 0.374 | 0.050 |
| Condition*age | 0.998 | 0.035 | 1 | 16 | 0.854 | 0.002 |
| Condition*UPDRS | 0.919 | 1.411 | 1 | 16 | 0.252 | 0.081 |
| Condition*disease duration | 0.975 | 0.407 | 1 | 16 | 0.532 | 0.025 |
| ROI*condition | 0.849 | 1.332 | 2 | 15 | 0.294 | 0.151 |
| ROI*condition*age | 0.816 | 1.689 | 2 | 15 | 0.218 | 0.184 |
| ROI*condition*UPDRS | 0.895 | 0.876 | 2 | 15 | 0.437 | 0.105 |
| ROI*condition*disease duration | 0.994 | 0.043 | 2 | 15 | 0.958 | 0.006 |
| ROI*modulation type | **0.372** | **12.648** | **2** | **15** | **<0.001** | **0.628** |
| ROI*modulation type*age | 0.963 | 0.292 | 2 | 15 | 0.751 | 0.037 |
| ROI*modulation type*UPDRS | 0.821 | 1.636 | 2 | 15 | 0.228 | 0.179 |
| ROI*modulation type*disease duration | 0.888 | 0.949 | 2 | 15 | 0.409 | 0.112 |
| Modulation type type*condition | 0.990 | 0.161 | 1 | 16 | 0.693 | 0.010 |
| Modulation type type*condition*age | 0.998 | 0.028 | 1 | 16 | 0.870 | 0.002 |
| Modulation type type*condition*UPDRS | 0.968 | 0.524 | 1 | 16 | 0.480 | 0.032 |
| Modulation type type*condition*disease duration | 0.919 | 1.411 | 1 | 16 | 0.252 | 0.081 |
| ROI*condition*  modulation type | 0.770 | 2.237 | 2 | 15 | 0.141 | 0.230 |
| ROI*condition*  modulation type*age | 0.712 | 3.035 | 2 | 15 | 0.078 | 0.288 |
| ROI*condition*  modulation type*UPDRS | 0.970 | 0.231 | 2 | 15 | 0.796 | 0.030 |
| ROI*condition*  modulation type*disease  duration | 0.770 | 2.239 | 2 | 15 | 0.141 | 0.230 |
