# Supplementary material for: Context-dependent modulations of subthalamo-cortical synchronization during rapid reversals of movement direction in Parkinson’s disease
Source: eLife. 2025 Jun 5;13:RP101769. doi: 10.7554/eLife.101769 (PMC12140627; doi:10.7554/eLife.101769)
Supplement: Supplementary file 3. — (A) Effects of condition (predictable, unpredictable), movement (start, reverse, stop), and regions of interest ROI (contralateral and ipsilateral STN, M1, MSMC) on normalized power, controlling for movement speed, age, pre-operative UPDRS score, and disease duration. (B) Effects of condition (predictable, unpredictable), movement (start, reverse, stop), and ROI (contralateral STN-M1, contralateral STN-MSMC, ipsilateral STN-M1, ipsilateral STN-MSMC) on coherence modulation, controlling for movement speed, age, pre-operative UPDRS score, and disease duration. [file elife-101769-supp3.docx]

**Supplementary File 3: Effects on beta power and coherence.** (**A**) Effects of condition (predictable, unpredictable), movement (start, reverse, stop) and ROI (contralateral and ipsilateral STN, M1, MSMC) on normalized power, controlling for movement speed, age, pre-operative UPDRS score and disease duration. (**B**) Effects of condition (predictable, unpredictable), movement (start, reverse, stop) and ROI (contralateral STN-M1, contralateral STN-MSMC, ipsilateral STN-M1, ipsilateral STN-MSMC) on coherence modulation, controlling for movement speed, age, pre-operative UPDRS score and disease duration.

**A**

| Factor | Wilk’s Lambda | *F* | Hypothesis *df* | Error *df* | Sig. | η_p_^2^ |  |
| --- | --- | --- | --- | --- | --- | --- | --- |
| Condition | 0.938 | 0.992 | 1 | 15 | 0.335 | 0.062 |  |
| Condition*speed | 0.936 | 1.034 | 1 | 15 | 0.325 | 0.064 |  |
| Condition*age | 0.938 | 0.991 | 1 | 15 | 0.335 | 0.062 |  |
| Condition*UPDRS | 0.960 | 0.632 | 1 | 15 | 0.439 | 0.040 |  |
| Condition*disease duration | 0.951 | 0.777 | 1 | 15 | 0.392 | 0.049 |  |
| ROI | **0.239** | **6.988** | **5** | **11** | **0.004** | **0.761** |  |
| ROI*speed | 0.595 | 1.500 | 5 | 11 | 0.267 | 0.405 |  |
| ROI*age | 0.900 | 0.245 | 5 | 11 | 0.934 | 0.100 |  |
| ROI*UPDRS | 0.740 | 0.773 | 5 | 11 | 0.589 | 0.260 |  |
| ROI*disease duration | 0.898 | 0.250 | 5 | 11 | 0.931 | 0.102 |  |
| Movement | **0.111** | **56.281** | **2** | **14** | **<0.001** | **0.889** |  |
| Movement*speed | 0.832 | 1.414 | 2 | 14 | 0.276 | 0.168 |  |
| Movement*age | 0.952 | 0.355 | 2 | 14 | 0.707 | 0.048 |  |
| Movement*UPDRS | 0.968 | 0.228 | 2 | 14 | 0.799 | 0.032 |  |
| Movement*disease duration | 0.919 | 0.618 | 2 | 14 | 0.553 | 0.081 |  |
| ROI*condition | 0.832 | 0.446 | 5 | 11 | 0.808 | 0.168 |  |
| ROI*condition*speed | 0.830 | 0.450 | 5 | 11 | 0.805 | 0.170 |  |
| ROI*condition*age | | 0.800 | 0.550 | 5 | 11 | 0.736 | 0.200 |
| ROI*condition*UPDRS | | 0.715 | 0.876 | 5 | 11 | 0.528 | 0.285 |
| ROI*condition*disease duration | | 0.567 | 1.683 | 5 | 11 | 0.219 | 0.433 |
| ROI*movement | **0.119** | **4.444** | **10** | **6** | **0.041** | **0.881** |  |
| ROI*movement*speed | 0.368 | 1.031 | 10 | 6 | 0.508 | 0.632 |  |
| ROI*movement*age | **0.128** | **4.078** | **10** | **6** | **0.049** | **0.872** |  |
| ROI*movement*UPDRS | 0.331 | 1.212 | 10 | 6 | 0.424 | 0.669 |  |
| ROI*movement*disease duration | 0.494 | 0.616 | 10 | 6 | 0.763 | 0.506 |  |
| Condition*movement | **0.625** | **4.206** | **2** | **14** | **0.037** | **0.375** |  |
| Condition*movement*speed | 0.710 | 2.866 | 2 | 14 | 0.091 | 0.290 |  |
| Condition*movement*age | 0.938 | 0.463 | 2 | 14 | 0.639 | 0.062 |  |
| Condition*movement*UPDRS | 0.752 | 2.308 | 2 | 14 | 0.136 | 0.248 |  |
| Condition*movement*disease duration | 0.936 | 0.481 | 2 | 14 | 0.628 | 0.064 |  |
| ROI*condition*movement | **0.083** | **6.666** | **10** | **6** | **0.015** | **0.917** |  |
| ROI*condition*movement*  speed | 0.429 | 0.800 | 10 | 6 | 0.641 | 0.571 |  |
| ROI*condition*movement*  age | 0.177 | 2.769 | 10 | 6 | 0.110 | 0.823 |  |
| ROI*condition*movement*  UPDRS | 0.520 | 0.554 | 10 | 6 | 0.805 | 0.480 |  |
| ROI*condition*movement*  disease duration | 0.387 | 0.952 | 10 | 6 | 0.551 | 0.613 |  |

**B**

| Condition | 0.633 | 8.684 | 1 | 15 | 0.010 | 0.367 |
| --- | --- | --- | --- | --- | --- | --- |
| Condition*speed | 0.962 | 0.595 | 1 | 15 | 0.453 | 0.038 |
| Condition*age | 0.992 | 0.113 | 1 | 15 | 0.741 | 0.008 |
| Condition*UPDRS | 0.942 | 0.929 | 1 | 15 | 0.350 | 0.058 |
| Condition*disease duration | 0.772 | 4.427 | 1 | 15 | 0.053 | 0.228 |
| ROI | **0.453** | **5.239** | **3** | **13** | **0.014** | **0.547** |
| ROI*speed | 0.717 | 1.714 | 3 | 13 | 0.213 | 0.283 |
| ROI*age | 0.682 | 2.017 | 3 | 13 | 0.161 | 0.318 |
| ROI*UPDRS | 0.977 | 0.100 | 3 | 13 | 0.959 | 0.023 |
| ROI*disease duration | 0.782 | 1.211 | 3 | 13 | 0.345 | 0.218 |
| Movement | **0.370** | **11.907** | **2** | **14** | **<0.001** | **0.630** |
| Movement*speed | 0.959 | 0.296 | 2 | 14 | 0.749 | 0.041 |
| Movement*age | 0.825 | 1.486 | 2 | 14 | 0.260 | 0.175 |
| Movement*UPDRS | 0.979 | 0.150 | 2 | 14 | 0.862 | 0.021 |
| Movement*disease duration | 0.991 | 0.061 | 2 | 14 | 0.941 | 0.009 |
| ROI*condition | 0.698 | 1.871 | 3 | 13 | 0.184 | 0.302 |
| ROI*condition*speed | 0.988 | 0.050 | 3 | 13 | 0.984 | 0.012 |
| ROI*condition*age | 0.892 | 0.526 | 3 | 13 | 0.672 | 0.108 |
| ROI*condition*UPDRS | 0.819 | 0.960 | 3 | 13 | 0.441 | 0.181 |
| ROI*condition*disease duration | 0.737 | 1.546 | 3 | 13 | 0.250 | 0.263 |
| ROI*movement | 0.518 | 1.548 | 6 | 10 | 0.258 | 0.482 |
| ROI*movement*speed | 0.457 | 1.982 | 6 | 10 | 0.162 | 0.543 |
| ROI*movement*age | 0.810 | 0.390 | 6 | 10 | 0.870 | 0.190 |
| ROI*movement*UPDRS | 0.619 | 1.026 | 6 | 10 | 0.462 | 0.381 |
| ROI*movement*disease duration | 0.528 | 1.487 | 6 | 10 | 0.276 | 0.472 |
| Condition*movement | 0.956 | 0.319 | 2 | 14 | 0.732 | 0.044 |
| Condition*movement  *speed | 0.939 | 0.453 | 2 | 14 | 0.644 | 0.061 |
| Condition*movement*age | 0.888 | 0.880 | 2 | 14 | 0.436 | 0.112 |
| Condition*movement*UPDRS | 0.925 | 0.565 | 2 | 14 | 0.581 | 0.075 |
| Condition*movement*disease duration | 0.817 | 1.572 | 2 | 14 | 0.242 | 0.183 |
| ROI*condition*movement | 0.642 | 0.930 | 6 | 10 | 0.513 | 0.358 |
| ROI*condition*movement  *speed | 0.614 | 1.048 | 6 | 10 | 0.451 | 0.386 |
| ROI*condition*movement*  age | 0.777 | 0.479 | 6 | 10 | 0.810 | 0.223 |
| ROI*condition*movement*  UPDRS | 0.819 | 0.367 | 6 | 10 | 0.884 | 0.181 |
| ROI*condition*movement*  disease duration | 0.484 | 1.780 | 6 | 10 | 0.201 | 0.516 |
