# Supplementary material for: Context-dependent modulations of subthalamo-cortical synchronization during rapid reversals of movement direction in Parkinson’s disease
Source: eLife. 2025 Jun 5;13:RP101769. doi: 10.7554/eLife.101769 (PMC12140627; doi:10.7554/eLife.101769)
Supplement: Supplementary file 4. — (A) Effects of condition (predictable, unpredictable), movement (start, reverse, stop), and regions of interest (ROI) (contralateral and ipsilateral M1->STN, STN->M1, MSMC->STN, STN->MSMC) on Granger causality, controlling for movement speed, age, pre-operative UPDRS score, and disease duration. [file elife-101769-supp4.docx]

**Supplementary File 4: Effects on beta granger causality.** (**A**) Effects of condition (predictable, unpredictable), movement (start, reverse, stop) and ROI (contralateral and ipsilateral M1->STN, STN->M1, MSMC->STN, STN->MSMC) on Granger causality, controlling for movement speed, age, pre-operative UPDRS score and disease duration.

**A**

| Factor | Wilk’s Lambda | *F* | Hypothesis *df* | Error *df* | Sig. | η_p_^2^ |
| --- | --- | --- | --- | --- | --- | --- |
| Condition | 0.911 | 1.459 | 1 | 15 | 0.246 | 0.089 |
| Condition*speed | 0.824 | 3.199 | 1 | 15 | 0.094 | 0.176 |
| Condition*age | 0.999 | 0.008 | 1 | 15 | 0.931 | 0.001 |
| Condition*UPDRS | 1.000 | 0.000 | 1 | 15 | 0.999 | 0.000 |
| Condition*disease duration | 0.998 | 0.023 | 1 | 15 | 0.881 | 0.002 |
| ROI | **0.272** | **3.443** | **7** | **9** | **0.044** | **0.728** |
| ROI*speed | 0.663 | 0.653 | 7 | 9 | 0.707 | 0.337 |
| ROI*age | 0.414 | 1.820 | 7 | 9 | 0.198 | 0.586 |
| ROI*UPDRS | 0.878 | 0.178 | 7 | 9 | 0.983 | 0.122 |
| ROI*disease duration | 0.590 | 0.893 | 7 | 9 | 0.549 | 0.410 |
| Movement | 0.774 | 2.045 | 2 | 14 | 0.166 | 0.226 |
| Movement*speed | 0.662 | 3.567 | 2 | 14 | 0.056 | 0.338 |
| Movement*age | 0.795 | 1.805 | 2 | 14 | 0.201 | 0.205 |
| Movement*UPDRS | 0.745 | 2.398 | 2 | 14 | 0.127 | 0.255 |
| Movement*disease duration | 0.990 | 0.072 | 2 | 14 | 0.931 | 0.010 |
| ROI*condition | 0.753 | 0.421 | 7 | 9 | 0.866 | 0.247 |
| ROI*condition*speed | 0.633 | 0.745 | 7 | 9 | 0.643 | 0.367 |
| ROI*condition*age | 0.741 | 0.450 | 7 | 9 | 0.848 | 0.259 |
| ROI*condition*UPDRS | 0.805 | 0.312 | 7 | 9 | 0.931 | 0.195 |
| ROI*condition*disease duration | 0.663 | 0.652 | 7 | 9 | 0.707 | 0.337 |
| ROI*movement | 0.062 | 2.178 | 14 | 2 | 0.359 | 0.938 |
| ROI*movement*speed | 0.150 | 0.808 | 14 | 2 | 0.680 | 0.850 |
| ROI*movement*age | 0.131 | 0.946 | 14 | 2 | 0.627 | 0.869 |
| ROI*movement*UPDRS | 0.232 | 0.474 | 14 | 2 | 0.842 | 0.768 |
| ROI*movement*disease duration | 0.139 | 0.887 | 14 | 2 | 0.648 | 0.861 |
| Condition*movement | 0.727 | 2.632 | 2 | 14 | 0.107 | 0.273 |
| Condition*movement  *speed | 0.798 | 1.767 | 2 | 14 | 0.207 | 0.202 |
| Condition*movement*age | 0.658 | 3.638 | 2 | 14 | 0.053 | 0.342 |
| Condition*movement*UPDRS | 0.955 | 0.333 | 2 | 14 | 0.722 | 0.045 |
| Condition*movement*disease duration | 0.898 | 0.794 | 2 | 14 | 0.471 | 0.102 |
| ROI*condition*movement | 0.078 | 1.698 | 14 | 2 | 0.432 | 0.922 |
| ROI*condition*movement  *speed | 0.149 | 0.816 | 14 | 2 | 0.677 | 0.851 |
| ROI*condition*movement*  age | 0.133 | 0.931 | 14 | 2 | 0.632 | 0.867 |
| ROI*condition*movement*  UPDRS | 0.157 | 0.766 | 14 | 2 | 0.698 | 0.843 |
| ROI*condition*movement*  disease duration | 0.106 | 1.200 | 14 | 2 | 0.545 | 0.894 |
