# Supplementary material for: Context-dependent modulations of subthalamo-cortical synchronization during rapid reversals of movement direction in Parkinson’s disease
Source: eLife. 2025 Jun 5;13:RP101769. doi: 10.7554/eLife.101769 (PMC12140627; doi:10.7554/eLife.101769)
Supplement: Supplementary file 5. — (A) Effects of condition (predictable, unpredictable), movement (start, reverse, stop), and regions of interest (ROI) (contralateral and ipsilateral STN, M1, MSMC) on normalized power, controlling for movement speed, age, pre-operative UPDRS score and disease duration. (B) Effects of condition (predictable, unpredictable), movement (start, reverse, stop), and ROI (contralateral STN-M1, contralateral STN-MSMC, ipsilateral STN-M1, ipsilateral STN-MSMC) on coherence modulation, controlling for movement speed, age, pre-operative UPDRS score, and disease duration. [file elife-101769-supp5.docx]

**Supplementary File 5: Effects on gamma power and coherence.** (**A**) Effects of condition (predictable, unpredictable), movement (start, reverse, stop) and ROI (contralateral and ipsilateral STN, M1, MSMC) on normalized power, controlling for movement speed, age, pre-operative UPDRS score and disease duration. (**B**) Effects of condition (predictable, unpredictable), movement (start, reverse, stop) and ROI (contralateral STN-M1, contralateral STN-MSMC, ipsilateral STN-M1, ipsilateral STN-MSMC) on coherence modulation, controlling for movement speed, age, pre-operative UPDRS score and disease duration.

**A**

| Factor | Wilk’s Lambda | *F* | Hypothesis *df* | Error *df* | Sig. | η_p_^2^ |  |
| --- | --- | --- | --- | --- | --- | --- | --- |
| Condition | 0.950 | 0.792 | 1 | 15 | 0.388 | 0.050 |  |
| Condition*speed | 0.849 | 2.667 | 1 | 15 | 0.123 | 0.151 |  |
| Condition*age | 0.998 | 0.032 | 1 | 15 | 0.861 | 0.002 |  |
| Condition*UPDRS | 0.904 | 1.592 | 1 | 15 | 0.226 | 0.096 |  |
| Condition*disease duration | 0.929 | 1.145 | 1 | 15 | 0.302 | 0.071 |  |
| ROI | 0.440 | 2.789 | 5 | 11 | 0.072 | 0.560 |  |
| ROI*speed | 0.593 | 1.510 | 5 | 11 | 0.264 | 0.407 |  |
| ROI*age | 0.788 | 0.592 | 5 | 11 | 0.707 | 0.212 |  |
| ROI*UPDRS | 0.807 | 0.526 | 5 | 11 | 0.753 | 0.193 |  |
| ROI*disease duration | **0.290** | **5.276** | **5** | **11** | **0.010** | **0.710** |  |
| Movement | **0.607** | **4.537** | **2** | **14** | **0.030** | **0.393** |  |
| Movement*speed | 0.966 | 0.247 | 2 | 14 | 0.784 | 0.034 |  |
| Movement*age | 0.925 | 0.567 | 2 | 14 | 0.580 | 0.075 |  |
| Movement*UPDRS | 0.940 | 0.450 | 2 | 14 | 0.647 | 0.060 |  |
| Movement*disease duration | 0.852 | 1.215 | 2 | 14 | 0.326 | 0.148 |  |
| ROI*condition | 0.745 | 0.752 | 5 | 11 | 0.602 | 0.255 |  |
| ROI*condition*speed | 0.598 | 1.447 | 5 | 11 | 0.273 | 0.402 |  |
| ROI*condition*age | | 0.864 | 0.347 | 5 | 11 | 0.874 | 0.136 |
| ROI*condition*UPDRS | | 0.645 | 1.212 | 5 | 11 | 0.366 | 0.355 |
| ROI*condition*disease duration | | 0.766 | 0.671 | 5 | 11 | 0.654 | 0.234 |
| ROI*movement | 0.163 | 3.073 | 10 | 6 | 0.091 | 0.837 |  |
| ROI*movement*speed | 0.389 | 0.944 | 10 | 6 | 0.555 | 0.611 |  |
| ROI*movement*age | 0.227 | 2.045 | 10 | 6 | 0.197 | 0.773 |  |
| ROI*movement*UPDRS | 0.537 | 0.518 | 10 | 6 | 0.829 | 0.463 |  |
| ROI*movement*disease duration | 0.234 | 1.962 | 10 | 6 | 0.212 | 0.766 |  |
| Condition*movement | 0.962 | 0.276 | 2 | 14 | 0.763 | 0.038 |  |
| Condition*movement*speed | 0.916 | 0.639 | 2 | 14 | 0.542 | 0.084 |  |
| Condition*movement*age | 0.992 | 0.054 | 2 | 14 | 0.947 | 0.008 |  |
| Condition*movement*UPDRS | 0.991 | 0.063 | 2 | 14 | 0.939 | 0.009 |  |
| Condition*movement*disease duration | 0.978 | 0.160 | 2 | 14 | 0.853 | 0.022 |  |
| ROI*condition*movement | 0.316 | 1.301 | 10 | 6 | 0.389 | 0.684 |  |
| ROI*condition*movement*  speed | 0.386 | 0.953 | 10 | 6 | 0.550 | 0.614 |  |
| ROI*condition*movement*  age | **0.041** | **14.067** | **10** | **6** | **0.002** | **0.959** |  |
| ROI*condition*movement*  UPDRS | **0.083** | **6.643** | **10** | **6** | **0.015** | **0.917** |  |
| ROI*condition*movement*  disease duration | 0.475 | 0.662 | 10 | 6 | 0.731 | 0.525 |  |

**B**

| Condition | 1.000 | 0.005 | 1 | 15 | 0.944 | 0.000 |
| --- | --- | --- | --- | --- | --- | --- |
| Condition*speed | 0.949 | 0.804 | 1 | 15 | 0.384 | 0.051 |
| Condition*age | 1.000 | 0.002 | 1 | 15 | 0.969 | 0.000 |
| Condition*UPDRS | 0.997 | 0.040 | 1 | 15 | 0.844 | 0.003 |
| Condition*disease duration | 0.983 | 0.257 | 1 | 15 | 0.619 | 0.017 |
| ROI | 0.921 | 0.371 | 3 | 13 | 0.775 | 0.079 |
| ROI*speed | 0.812 | 1.006 | 3 | 13 | 0.422 | 0.188 |
| ROI*age | 0.956 | 0.200 | 3 | 13 | 0.894 | 0.044 |
| ROI*UPDRS | 0.914 | 0.407 | 3 | 13 | 0.751 | 0.086 |
| ROI*disease duration | 0.721 | 1.675 | 3 | 13 | 0.221 | 0.279 |
| Movement | 0.687 | 3.195 | 2 | 14 | 0.072 | 0.313 |
| Movement*speed | 0.918 | 0.626 | 2 | 14 | 0.549 | 0.082 |
| Movement*age | 0.819 | 1.547 | 2 | 14 | 0.247 | 0.181 |
| Movement*UPDRS | **0.645** | **3.851** | **2** | **14** | **0.047** | **0.355** |
| Movement*disease duration | 0.920 | 0.605 | 2 | 14 | 0.560 | 0.080 |
| ROI*condition | 0.809 | 1.025 | 3 | 13 | 0.414 | 0.191 |
| ROI*condition*speed | 0.944 | 0.256 | 3 | 13 | 0.856 | 0.056 |
| ROI*condition*age | 0.764 | 1.339 | 3 | 13 | 0.305 | 0.236 |
| ROI*condition*UPDRS | 0.929 | 0.330 | 3 | 13 | 0.804 | 0.071 |
| ROI*condition*disease duration | 0.562 | 3.373 | 3 | 13 | 0.051 | 0.438 |
| ROI*movement | 0.569 | 1.263 | 6 | 10 | 0.354 | 0.431 |
| ROI*movement*speed | 0.870 | 0.248 | 6 | 10 | 0.949 | 0.130 |
| ROI*movement*age | 0.976 | 0.042 | 6 | 10 | 1.000 | 0.024 |
| ROI*movement*UPDRS | 0.788 | 0.448 | 6 | 10 | 0.831 | 0.212 |
| ROI*movement*disease duration | 0.502 | 1.655 | 6 | 10 | 0.230 | 0.498 |
| Condition*movement | 0.681 | 3.274 | 2 | 14 | 0.068 | 0.319 |
| Condition*movement  *speed | 0.852 | 1.214 | 2 | 14 | 0.326 | 0.148 |
| Condition*movement*age | 0.808 | 1.665 | 2 | 14 | 0.225 | 0.192 |
| Condition*movement*UPDRS | 0.908 | 0.709 | 2 | 14 | 0.509 | 0.092 |
| Condition*movement*disease duration | 0.937 | 0.467 | 2 | 14 | 0.636 | 0.063 |
| ROI*condition*movement | 0.641 | 0.935 | 6 | 10 | 0.511 | 0.359 |
| ROI*condition*movement  *speed | 0.659 | 0.862 | 6 | 10 | 0.553 | 0.341 |
| ROI*condition*movement*  age | 0.554 | 1.344 | 6 | 10 | 0.323 | 0.446 |
| ROI*condition*movement*  UPDRS | 0.650 | 0.897 | 6 | 10 | 0.532 | 0.350 |
| ROI*condition*movement*  disease duration | 0.632 | 1.972 | 6 | 10 | 0.490 | 0.368 |
