# Supplementary material for: Context-dependent modulations of subthalamo-cortical synchronization during rapid reversals of movement direction in Parkinson’s disease
Source: eLife. 2025 Jun 5;13:RP101769. doi: 10.7554/eLife.101769 (PMC12140627; doi:10.7554/eLife.101769)
Supplement: Supplementary file 6. — (A) Effects of condition (predictable, unpredictable), movement (start, reverse, stop), and regions of interest (ROI) (contralateral and ipsilateral M1->STN, STN->M1, MSMC->STN, STN->MSMC) on Granger causality, controlling for movement speed, age, pre-operative UPDRS score, and disease duration. [file elife-101769-supp6.docx]

**Supplementary File 6: Effects on gamma granger causality.** (**A**) Effects of condition (predictable, unpredictable), movement (start, reverse, stop) and ROI (contralateral and ipsilateral M1->STN, STN->M1, MSMC->STN, STN->MSMC) on Granger causality, controlling for movement speed, age, pre-operative UPDRS score and disease duration.

**A**

| Factor | Wilk’s Lambda | *F* | Hypothesis *df* | Error *df* | Sig. | η_p_^2^ |
| --- | --- | --- | --- | --- | --- | --- |
| Condition | 0.814 | 3.435 | 1 | 15 | 0.084 | 0.186 |
| Condition*speed | 0.995 | 0.077 | 1 | 15 | 0.786 | 0.005 |
| Condition*age | 0.999 | 0.015 | 1 | 15 | 0.904 | 0.001 |
| Condition*UPDRS | 0.989 | 0.164 | 1 | 15 | 0.691 | 0.011 |
| Condition*disease duration | 0.984 | 0.238 | 1 | 15 | 0.633 | 0.016 |
| ROI | 0.792 | 0.338 | 7 | 9 | 0.917 | 0.208 |
| ROI*speed | 0.372 | 2.170 | 7 | 9 | 0.138 | 0.628 |
| ROI*age | 0.590 | 0.893 | 7 | 9 | 0.549 | 0.410 |
| ROI*UPDRS | 0.539 | 1.098 | 7 | 9 | 0.437 | 0.461 |
| ROI*disease duration | 0.773 | 0.378 | 7 | 9 | 0.894 | 0.227 |
| Movement | 0.774 | 2.043 | 2 | 14 | 0.167 | 0.226 |
| Movement*speed | 0.812 | 1.620 | 2 | 14 | 0.233 | 0.188 |
| Movement*age | 0.950 | 0.371 | 2 | 14 | 0.697 | 0.050 |
| Movement*UPDRS | 0.824 | 1.497 | 2 | 14 | 0.258 | 0.176 |
| Movement*disease duration | 0.966 | 0.245 | 2 | 14 | 0.786 | 0.034 |
| ROI*condition | 0.712 | 0.520 | 7 | 9 | 0.800 | 0.288 |
| ROI*condition*speed | 0.641 | 0.720 | 7 | 9 | 0.660 | 0.359 |
| ROI*condition*age | 0.541 | 1.090 | 7 | 9 | 0.441 | 0.459 |
| ROI*condition*UPDRS | 0.541 | 1.089 | 7 | 9 | 0.442 | 0.459 |
| ROI*condition*disease duration | 0.758 | 0.411 | 7 | 9 | 0.873 | 0.242 |
| ROI*movement | 0.039 | 3.478 | 14 | 2 | 0.246 | 0.961 |
| ROI*movement*speed | 0.133 | 0.932 | 14 | 2 | 0.631 | 0.867 |
| ROI*movement*age | 0.204 | 0.556 | 14 | 2 | 0.798 | 0.796 |
| ROI*movement*UPDRS | 0.250 | 0.428 | 14 | 2 | 0.867 | 0.750 |
| ROI*movement*disease duration | 0.149 | 0.813 | 14 | 2 | 0.678 | 0.851 |
| Condition*movement | 0.665 | 3.519 | 2 | 14 | 0.058 | 0.335 |
| Condition*movement  *speed | 0.955 | 0.333 | 2 | 14 | 0.722 | 0.045 |
| Condition*movement*age | 0.791 | 1.846 | 2 | 14 | 0.194 | 0.209 |
| Condition*movement*UPDRS | 0.894 | 0.831 | 2 | 14 | 0.456 | 0.106 |
| Condition*movement*disease duration | 0.983 | 0.119 | 2 | 14 | 0.889 | 0.017 |
| ROI*condition*movement | 0.191 | 0.604 | 14 | 2 | 0.774 | 0.809 |
| ROI*condition*movement  *speed | 0.157 | 0.768 | 14 | 2 | 0.697 | 0.843 |
| ROI*condition*movement*  age | 0.314 | 0.313 | 14 | 2 | 0.928 | 0.686 |
| ROI*condition*movement*  UPDRS | 0.402 | 0.212 | 14 | 2 | 0.973 | 0.598 |
| ROI*condition*movement*  disease duration | 0.084 | 1.565 | 14 | 2 | 0.458 | 0.916 |
